# Statin-induced depletion of geranylgeranyl pyrophosphate inhibits cell proliferation by a novel pathway of Skp2 degradation

**DOI:** 10.18632/oncotarget.3068

**Published:** 2014-12-26

**Authors:** Jonathan Vosper, Alessia Masuccio, Michael Kullmann, Christian Ploner, Stephan Geley, Ludger Hengst

**Affiliations:** ^1^ Division of Medical Biochemistry, Biocenter, Medical University of Innsbruck, Innsbruck, Austria; ^2^ Division of Molecular Pathophysiology, Biocenter/Clinic of Plastic and Reconstructive Surgery, Medical University of Innsbruck, Innsbruck, Austria; ^3^ Division of Molecular Pathophysiology, Biocenter, Medical University of Innsbruck, Innsbruck, Austria

**Keywords:** cell cycle, geranylgeranylation, lovastatin, p27, Skp2

## Abstract

Statins, such as lovastatin, can induce a cell cycle arrest in the G1 phase. This robust antiproliferative activity remains intact in many cancer cells that are deficient in cell cycle checkpoints and leads to an increased expression of CDK inhibitor proteins p27^Kip1^ and p21^Cip1^. The molecular details of this statin-induced growth arrest remains unclear. Here we present evidence that lovastatin can induce the degradation of Skp2, a subunit of the SCF^Skp2^ ubiquitin ligase that targets p27^Kip1^ and p21^Cip1^ for proteasomal destruction.

The statin-induced degradation of Skp2 is cell cycle phase independent and does not require its well characterised degradation pathway mediated by APC/C^Cdh1-^ or Skp2 autoubiquitination. An N-terminal domain preceding the F-box of Skp2 is both necessary and sufficient for its statin mediated degradation. The degradation of Skp2 results from statin induced depletion of geranylgeranyl isoprenoid intermediates of cholesterol biosynthesis. Inhibition of geranylgeranyl-transferase-I also promotes APC/C^Cdh1^-independent degradation of Skp2, indicating that de-modification of a geranylgeranylated protein triggers this novel pathway of Skp2 degradation.

## INTRODUCTION

Statins are a class of small fungal metabolites that inhibit the HMG-CoA reductase, an enzyme that catalyses the reduction of 3-hydroxy-3-methyl-glutaryl-CoA (HMG-CoA) to mevalonate [1]. This is a rate-limiting step of the cholesterol biosynthesis pathway. For this reason statins have been used extensively for the treatment of hypercholesterolemia. It has been appreciated for some time that statins also can induce growth arrest in several diverse cancer cell lines [2, 3]. Statin induced apoptosis has also been reported to occur in a subset of tumour cell types [3]. Importantly, normal cells appear to be more resistant to statin induced growth arrest and cytotoxicity. Thus statins have been reported to inhibit proliferation of karyotipically abnormal embryonic stem cells but not normal embryonic stem cells [4]. In addition, lipophilic statins were found to induce cell death in MCF-7 cells more efficiently than in non-cancerous MCF-10 epithelial cells [5]. *In vivo*, lovastatin has also been shown to prevent carcinogenesis in a rat model for liver cancer [6]. Furthermore, recent evidence indicates that statins can impinge on cancer development at different stages of progression. Thus statins have the potential to interfere with the invasion and migration of prostate cancer cells via negative regulation of phospholipase 2 group VII (PLA2G7) [7]. Interestingly, statins have also been shown to have an immunomodulatory function in spontaneous mouse mammary tumours by promoting T cell infiltration [8]. This activity was shown to lead to reduced formation of new lesions in a mouse genetic model for tumour development. Prospective observational studies in humans revealed a significant reduction in cancer risk (14%-28%) associated with Statin use [9].

These observations coupled with a well-established clinical history in the treatment of hypercholesterolemia have led to the proposition that statins may be used in the treatment of cancer. However, conclusive data from randomised control trials for statin use and cancer risk are currently lacking. In general statin treatment seems to be beneficial for the treatment of certain types of cancer but not for others [10]. Statins also target a broad range of cellular processes which may confound their efficacy [11]. Therefore a better understanding of the molecular pathways responsible for their ability to inhibit cell proliferation seems important.

Statins can lead to the induction of the cyclin dependent kinase inhibitors (CKI) p27^Kip1^ and p21^Cip1^, leading to decreased cyclin dependent kinase activity and subsequent cell cycle arrest in G1 phase [12-14]. The induction of p27 by lovastatin is regulated post-transcriptionally [15]. During normal cell cycle progression, p27^Kip1^ is ubiquitinated in late G1 phase by the SCF^Skp2^ ubiquitin ligase and thereby targeted for proteasomal degradation [16-19]. SCF^Skp2^ is also an ubiquitin ligase for p21^Cip1^ [20, 21]. It has proposed that lovastatin can up regulate p27^Kip1^ and p21^Cip1^ by directly inhibiting the 26S proteasome [22]. The presence of a lactone moiety in the closed ring prodrug form of lovastatin that can directly inhibit the chymotrypsin like catalytic activity of the proteasome has been posited to account for this G1 arresting activity [22, 23]. However other reports demonstrate that neither the prodrug nor the active dihydroxy acid form of lovastatin can inhibit the proteasome in SSC25, HeLa or 293T cells [24]. Indeed, one study suggests that the closed ring form can mildly stimulate the chymotrypsin like activity of purified bovine 20S proteasomes [25].

We previously observed that the level of the Skp2 protein, a E3 ubiquitin ligase subunit, regulating p27 stability during the G1/S transition is specifically reduced significantly following lovastatin-induced cell cycle arrest, but not a cell cycle arrest induced by nocodazole or thymidine [26]. Skp2 is an oncogene that has been shown to be an independent marker for poor prognosis in several types of human cancer [27-29]. We wondered if the reduced Skp2 levels were causing the elevated levels of p27 in lovastatin treated cells and therefore driving cessation of proliferation. This could reconcile the contradictory reports on the effects of lovastatin on proteasome activity; rather than a general blockade of proteasome activity, we speculated that by down regulating Skp2, lovastatin might prevent the proteasomal degradation of p27 and p21, whilst not affecting proteasomal turnover in general.

Here we show that Skp2 is robustly and specifically down regulated in response to lovastatin treatment and that this precedes the p27 and p21 accumulation. Downregulation of Skp2 is cell cycle phase independent and therefore not a consequence of the cell cycle arrest in G1 phase. Furthermore, we establish that destabilisation of the protein is responsible for the downregulation of Skp2. Interestingly, the mechanism for degrading Skp2 is distinct from the previously reported Cdh1-dependent degradation or auto-ubiquitination [30-32], thus representing a novel pathway of Skp2 degradation. Lovastatin triggers this pathway by preventing the geranyl-geranylation of an as yet unknown protein, since geranylgeranyl pyrophosphate rescues the lovastatin arrest and inhibition of geranylgeranyl transferase I also induces downregulation of Skp2. An N-terminal domain in Skp2 is required and sufficient to permit its downregulation by lovastatin and inhibitors of geranylgeranyl transferases.

## RESULTS

### Lovastatin induces downregulation of the Skp2 protein, but does not alter Skp2 mRNA level

**Figure 1:**
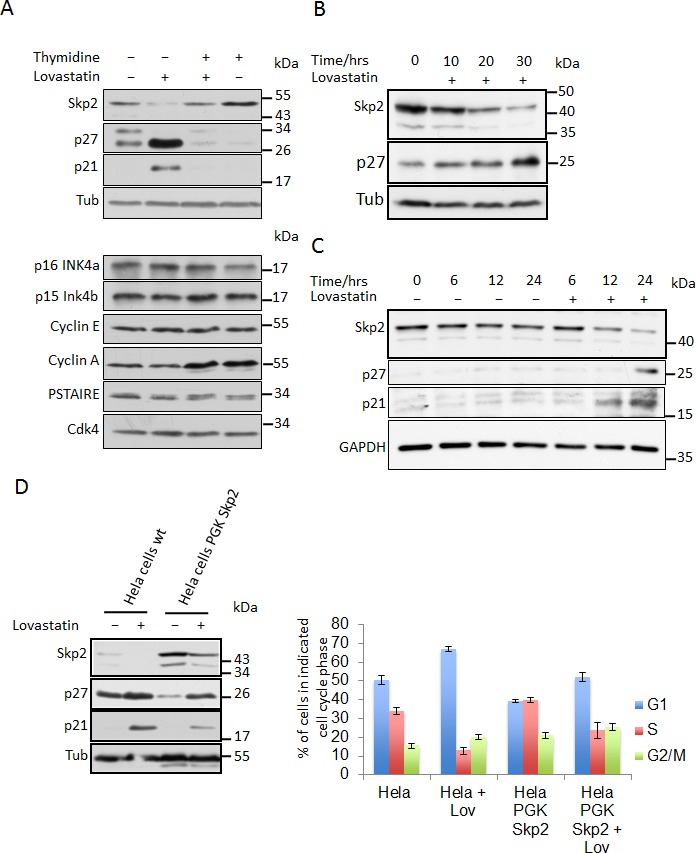

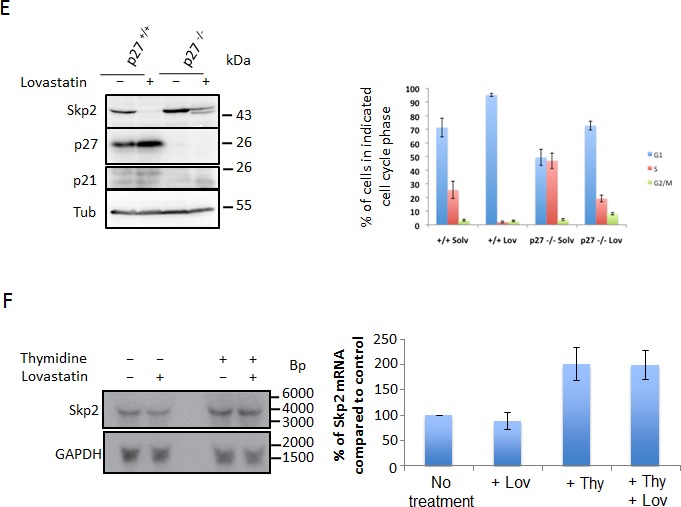
Lovastatin induces downregulation of the Skp2 protein (A) HeLa cells growing asynchronously or arrested in S-Phase by treatment with 2mM thymidine for 24 hours, were then treated or left untreated with 40μM lovastatin for a further 24 hours and analysed by (A) immunoblotting. (B) LnCAP and HeLa cells (C) were incubated with 10μM and 40μM lovastatin respectively for the indicated times and analysed by immunoblotting. (D) HeLa cells stably expressing Skp2 from a PGK promoter were generated by lentiviral transfection. These cells were treated with 40μM lovastatin for 24 hours and then subjected to immunoblotting analysis. Percentages of cells in the different cell cycle phases were determined by flow cytometric analysis of three independent experiments using propidium iodine and BrdU double staining (Bar graph; data are represented as mean +/− SEM). (E) Control MEFs and p27 ^−/−^ MEFs were treated with 20μM lovastatin for 24 hours as indicated, harvested and analysed by western blotting analysis (left panel). Flow cytometry analysis of BrdU labeled cells to determine cell cycle phase distribution was performed. The percentage of cells in G1, S or G2/M phase is shown for at least three independent experiments (right panel). (F) Skp2 mRNA analysis of HeLa cells treated as described in Fig. 1A. Northern blots from three independent experiments were quantified using a PhosphorImager and the relative abundance of the Skp2 mRNA is shown (bar graph; data are represented as mean +/− SEM).

As reported previously, treatment of cells with lovastatin leads to increased levels of p21 and p27 and an accumulation of cells in G1 phase of the cell cycle [12-14, 26, 33] (Fig. 1A, C). This is associated with significantly reduced Skp2 protein level, whereas the INK4 inhibitors p15 and p16, cyclins A and E as well as CDK level remain mostly unaltered (Fig. 1A). Since the level of Skp2 protein is normally low during early and mid G1 phase, the reduced amount of Skp2 protein following lovastatin treatment might be a consequence of the cell cycle arrest rather than its cause. Skp2 transcription and protein levels are increased in S-phase [32, 34]. To rule out a cell cycle positional effect, we first arrested HeLa cells in S-phase and subsequently treated the S-phase arrested cells with lovastatin. Lovastatin caused a clear decline in the Skp2 protein level even in S-phase arrested cells, where Skp2 usually is relatively stable (Fig. 1A, + thymidine), demonstrating that the reduction of Skp2 occurs independently of the G1 arrest caused by lovastatin. p21 and p27 did not accumulate in S-phase arrested cells, probably because Skp2 levels remain generally elevated and these remaining levels of Skp2 may be sufficient to promote p21 and p27 degradation. In fact, even though Skp2 levels in lovastatin-treated S-phase cells were significantly reduced, they remain similar to those of proliferating (untreated) cells. Thus the decrease in Skp2 protein following lovastatin treatment does not merely reflect the cell cycle position, but suggests a cell cycle independent direct pathway regulating Skp2 abundance upon lovastatin addition. This could cause the stabilisation of p27 and p21 and the subsequent cell cycle arrest.

Consistent with this model, we observed that the lovastatin-induced decrease in Skp2 precedes the accumulation of p27 (Fig. 1B,C). To directly investigate the hypothesis that Skp2 decline is driving accumulation of p27 and subsequent inhibition of proliferation, we generated virally transduced cell lines stably overexpressing the Skp2 cDNA. As reported previously, Skp2 overexpression is able to induce degradation of p27 [18, 26]. p27 is known to be phosphorylated on threonine residue 187 in lovastatin-arrested cells [18, 26], generating the phosphodegron which is a prerequisite for its SCF-initiated degradation. Overexpression of Skp2 reduced p27 levels in stably transduced cells in the presence and absence of lovastatin. Importantly, Skp2 overexpression could attenuate the lovastatin-induced accumulation of the CDK inhibitors with p27 level similar to those of proliferating cells (Fig. 1D). This corresponded with a decreased number of cells in G1-phase and increased number of cells in S-phase as revealed by FACS analysis (Fig. 1D), consistent with the hypothesis that the decline of Skp2 promotes the accumulation of p21 and p27 in lovastatin arrested cells. As a main substrate of Skp2 is the CDK inhibitor p27 [19], we investigated if cells lacking p27 expression are less sensitive to lovastatin-induced cell cycle arrest. Immortalised p27^−/−^ mouse embryonic fibroblasts were incubated with lovastatin for 24 hrs and compared to wt MEFs. Untreated p27^−/−^ MEFs are characterised by reduced cells in G1 phase and increased cells in S-phase (Fig. 1E). Upon incubation in lovastatin, less p27^−/−^ cells accumulated in G1 phase (73% vs. 95%) and more cells remained in S-phase (19% of p27^−/−^ compared to 2% of wt MEFs). These data further support our hypothesis that the Skp2-p27 axis plays an important role in the lovastatin-induced cell cycle arrest.

Of note, statins are also able to induce a decline of overexpressed Skp2. This suggests that lovastatin regulates Skp2 levels independently of transcription from the endogenous *Skp2* gene, since overexpression was achieved by viral transduction of a Skp2 cDNA expressed under the control of the phosphoglycerate kinase (PGK) promoter. To investigate if the reduction of Skp2 is transcriptionally regulated or due to altered Skp2 mRNA stability, we compared Skp2 mRNA levels in lovastatin-arrested cells and untreated cells. In contrast to the protein level, no significant reduction of Skp2 mRNA was observed in asynchronous cells as well as in S-phase arrested cells (Fig. 1F). This excludes transcriptional mechanisms and the regulation of Skp2 mRNA stability as a cause of the Skp2 elimination.

### Lovastatin induces degradation of Skp2 which is independent of APC/C^Cdh1^

**Figure 2:**
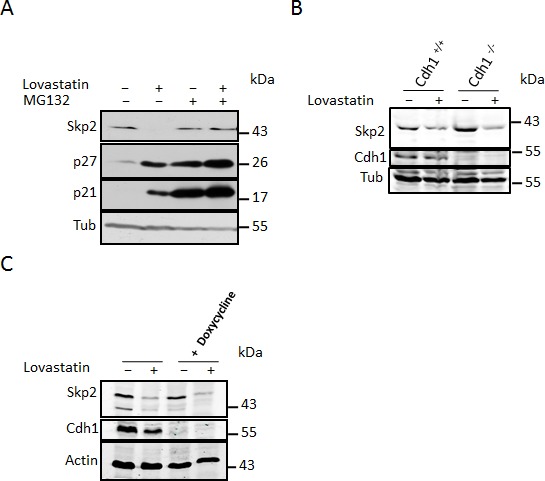
Lovastatin induces Skp2 protein degradation which is independent of its canonical E3 ubiquitin ligase APC/C^Cdhl^ (A) HeLa cells were treated with 40μM Lovastatin in the presence or absence of 10μM MG132. After 24 hours, samples were harvested and analysed by western blotting. (B) Control MEFs and Cdh1 ^−/−^ MEFs were treated with 40μM lovastatin for 24 hours, harvested and subjected to western blotting analysis. (C) Utsh2 cells in which Cdh1 can be inducibly knocked down were treated with or without 1μg/ml doxycycline for 48 hours in order to induce Cdh1 knockdown after which they were replated in medium containing 100ng/ml doxycycline and incubated for a further 24 hours and then supplemented with 40μM lovastatin for 48 hours after which they were harvested and subjected to Western blotting analysis.

Northern blot analysis demonstrated that the decrease in Skp2 levels is based on a post-transcriptional mechanism. The Skp2 protein itself is known to undergo ubiquitin-dependent proteasomal degradation. In order to investigate if lovastatin might induce the degradation of the Skp2 protein, we treated cells with the cell permeable reversible proteasome inhibitor MG132 (carbobenzoxy-Leu-Leu-leucinal) and lovastatin and monitored the Skp2 decline. The peptide aldehyde MG132 prevents down-regulation of Skp2 following lovastatin treatment (Fig. 2A). This demonstrates that inhibition of proteolysis restores Skp2 expression, suggesting that lovastatin initiates the degradation of the protein. As expected, MG132 also induced stabilisation of p21 and p27, which are substrates of the ubiquitin-proteasome system (Fig. 2A).

The stability of Skp2 is cell cycle regulated. Another ubiquitin E3 ligase complex, APC/C^Cdh1^ initiates its ubiquitin-mediated proteasomal degradation. We used mouse embryonic knockout fibroblasts for the APC subunit Cdh1 to determine if Skp2 degradation induced by lovastatin was dependent on the APC/C^Cdh1^ ubiquitin ligase. As can be seen in (Fig. 2B), lovastatin treatment results in a decrease in Skp2 levels even in the absence of Cdh1. This was also observed in cells in which Cdh1 can be inducibly knocked down by doxycycline (Fig. 2C). Since we can also exclude autoubiquitination as a mechanism triggering the Skp2 elimination induced by statins (see below), a novel pathway is responsible for the statin-induced degradation of Skp2.

### Skp2 downregulation is dependent on geranylgeranylation

**Figure 3:**
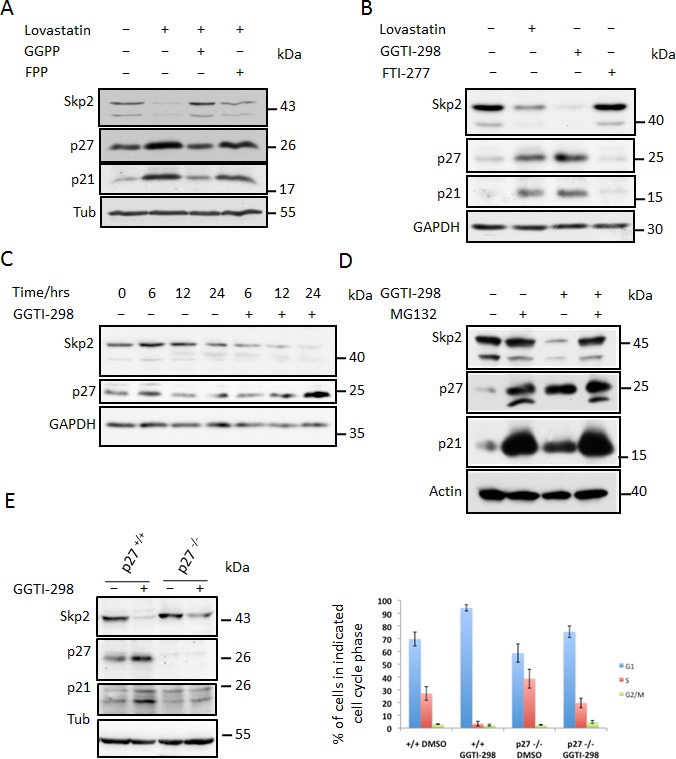

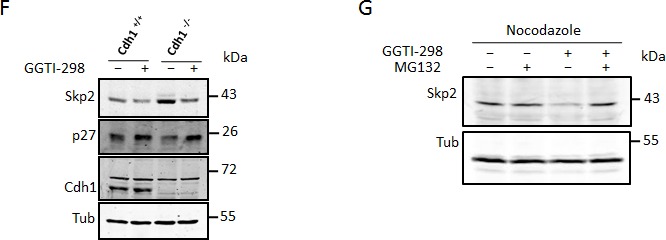
Skp2 decline is due to blockade of geranylgeranylation (A) HeLa cells were treated with 40μM lovastatin in the presence of geranylgeranylpyrophosphate (GGPP) or farnesylpyrophosphate (FPP). After 24 hours incubation, cells were harvested and subjected to western blotting analysis. (B) HeLa cells were treated 20μM lovastatin, geranylgeranyltransferase inhibitor GGTI-298 or farnesyltransferase inhibitor FTI-277 for 36 hours and analysed by western blotting. (C) HeLa cells were treated with 10μM GGTI-298 for different times (as indicated) after which Skp2, p27 levels were determined by immunoblot analysis. (D) HeLa cells were treated with 10μM GGTI-298 in the presence and absence of 20mM MG132. After 24 hours, samples were harvested and analysed by western blotting. (E) Control MEFs and p27 ^−/−^ MEFs were treated with 10 μM GGTI-298 for 24 hours as indicated, harvested and analysed by western blotting analysis (left panel). Flow cytometry analysis of BrdU labeled cells to determine cell cycle phase distribution was performed. The percentage of cells in G1, S or G2/M phase is shown for at least three independent experiments (right panel). (F) Control MEFs and Cdh1^−/−^ MEFs were treated with 10μM GGTI-298 for 24 hours then harvested and subjected to western blotting analysis. (G) Hela cells were treated with 50ng/ml Nocodazole for 18 hours after which they were supplemented with GGTI-298 at a final concentration of 10μM or solvent control. After a further 3 hours cells were supplemented with MG132 (20μM) or solvent and incubated for a further 9 hours. Cells were then harvested and processed for western blotting analysis.

Early intermediates of the cholesterol biosynthesis pathway such as mevalonate and isoprenoids can rescue statin mediated G1 arrest, whereas late intermediates such as squalene and cholesterol cannot [2]. Isoprenoid lipids are known to be required for the function of a number of proteins including oncogenic GTPases such as Ras and Rho proteins [35]. Furthermore, a number of studies have shown that inhibitors of isoprenylation can inhibit cell proliferation. We therefore tested the ability of geranylgeranyl pyrophosphate (GGPP) and farnesyl pyrophosphate (FPP) to prevent Skp2 down-regulation following lovastatin treatment. We found that GGPP could completely prevent the Skp2 decrease as well as the p27 and p21 increase, whereas FPP did not significantly alter the ability of lovastatin to down regulate Skp2 and increase p21 and p27 (Fig. 3A). As FPP is a precursor of GGPP, one might expect that FPP should also rescue the Lovastatin effect. However, in order to produce a 20 carbon geranylgeranylpyrophosphate from a 15 carbon FPP precursor, it requires the 5 carbon isopentenyl pyrophosphate. As lovastatin blocks the de novo synthesis of isopentyl pyrophosphate, which is synthesized from mevalonic acid, its depletion should inhibit the GGPP synthesis from FPP.

**Figure 4:**
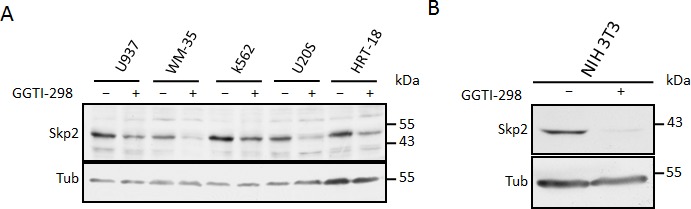
Inhibition of geranylgeranylation induces Skp2 degradation in many cell types (A) A selection of tumour dervied human cell lines were incubated with 10μM GGTI-298 after which Skp2 levels were determined by immunoblotting. (B) NIH3T3 cells were incubated with 10μM GGTI-298 for 24 hours after which Skp2 levels were determined by immunoblotting.

We speculated that inhibition of protein geranylgeranylation might cause the lovastatin induced Skp2 degradation. To investigate if blockade of protein geranylgeranylation might induce Skp2 degradation, we asked if inhibitors of geranylgeranylation could mimic the effects of lovastatin on Skp2. Inhibition of geranylgeranyltransferase I (GGTI) with the small molecule inhibitor GGTI-298 led to a dramatic decline in Skp2 levels and to an increase of p21 and p27 (Fig. 3B). In contrast, an inhibitor of farnesyltransferase, FTI-277, was not able to induce Skp2 down-regulation (Fig. 3B). These data indicate that lack of geranylgeranylation is responsible for the statin-induced Skp2 decline. Skp2 declined rapidly in response to GGTI-298 treatment with a significant decline occurring already at 6 hours of treatment (Fig. 3C). p27 accumulation commences after 12 hours and is clearly apparent after 24 hours. Thus the sequential order of Skp2 decline and p27 accumulation is conserved between GGTI-298 and lovastatin treatment. The Skp2 decline following GGTI-298 treatment is also prevented by MG132 treatment (Fig. 3D), supporting our model that lovastatin induces Skp2 degradation by inhibiting protein geranylgeranylation. As for lovastatin, knockout of p27 in mouse embryonic fibroblasts also attenuated G1 accumulation and inhibition of cell proliferation (Fig. 3E). Using the Cdh1^−/−^ MEFs, we found that the GGTI-298 induced Skp2 decline (like that of lovastatin) still occurs in the absence of Cdh1 (Fig. 3F). Using Nocodazole treatment preceding GGTI-298 incubation we could also demonstrate that GGTI-298 can induce proteolytic destruction of Skp2 in G2/M arrested cells (Fig. 3G), confirming that this degradation pathway is independent from a G1 arrest and active in different phases of the cell cycle. Together, these data indicate that lovastatin and GGTI-298 promote the specific degradation of Skp2 and that this enables p27 to accumulate. The inhibition of geranylgeranyltransferases with GGTI-298 uses a highly conserved and robust mechanism to downregulate Skp2, as we observed this downregulation in all human and murine cell lines tested (Fig. 4 A,B).

### The N-terminal domain of Skp2 is required for the down-regulation

**Figure 5:**
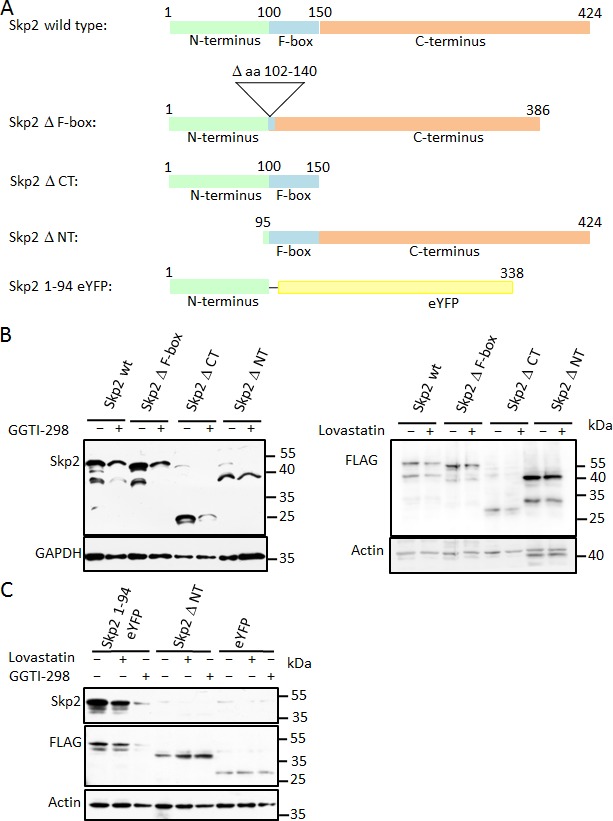
Skp2 degradation induced by GGTI-298/lovastatin depends on the N-terminal region which is sufficient to destabilise GFP in the presence of GGTI-298/lovastatin (A) Schematic representation of Skp2 functional domain mutants. (B) HeLa cells were transfected with the indicated FLAG-tagged Skp2 constructs and 24 hours after transfection incubated in the presence of 11μM GGTI-298 (left panel) of 60μM lovastatin (right panel) for 24 hours, after which they were analysed by western blotting. (C) A FLAG-tagged fusion protein consisting of the N-terminus of Skp2 fused to eYFP was transfected into HeLa cells and after 24 hours incubation cells were then treated with 40μM lovastatin or 10μM GGTI-298 as indicated for an additional 24 hours. FLAG-tagged Skp2 Δ NT and eYFP were transfected as controls. Cells were harvested and analysed by western blotting.

We next aimed to identify a domain responsible for the statin-mediated degradation of Skp2. To identify sequence elements of Skp2 required for its downregulation by lovastatin or GGTI-298, we analysed several point mutants and deletion mutants for their ability to undergo GGTI-298 or lovastatin induced degradation. Structural [36] and functional [16, 18, 37] analysis of Skp2 has identified at least three separate domains: A N-terminal regulatory domain, the F-box domain required for interaction with the core SCF subunit Skp1 [38] and a C-terminal leucine-rich repeat region forming a concave surface that interacts with phosphorylated substrate proteins [36, 37, 39]. We generated a Skp2 mutant lacking the internal F-box domain [16], a deletion mutant of the N-terminal domain, (lacking amino acids 1-94: Skp2 Δ NT), and a deletion mutant that lacks the C-terminal LRR domain (Skp2 Δ CT) (Fig. 5A). These mutants were transfected into HeLa cells, which were then subsequently treated with GGTI-298 or lovastatin. The F-box of Skp2 is essential for its incorporation into the SCF complex [38]. A mutant lacking this domain was still regulated by GGTI-298 and lovastatin, indicating that incorporation of Skp2 into an SCF is not a prerequisite for its lovastatin or GGTI-298 induced degradation (Fig. 5B). Since the F-box domain is also required for the auto-ubiquitination of Skp2 by the bound SCF core complex [30], and since p27 inhibits Skp2 auto-ubiquitination *in vivo* [40], it is unlikely that auto-ubiquitination is contributing to the lovastatin or GGTI-298 induced elimination of Skp2.

The mutant lacking the C-terminal domain was down regulated to a similar extent as the wt protein following GGTI-298 or lovastatin addition (Fig. 5B). In contrast, the mutant lacking the first 94 amino acids is significantly stabilised (Fig. 5B), even though it contains the F-box domain.

These data suggested that an important determinant for lovastatin/GGTI-298 mediated Skp2 destabilisation resides in the N-terminus of the molecule. We therefore investigated if this N-terminal region might be sufficient to initiate degradation triggered by GGTI-298 or lovastatin. We fused this N-terminal domain fragment to enhanced YFP (eYFP) (Fig. 5A). The fusion protein, Skp2 Δ NT and eYFP were expressed in HeLa cells. The levels of YFP alone or Skp2 lacking its N-terminus (Skp2 Δ NT) are not appreciably reduced by either lovastatin or GGTI-298 treatment. However, the first 94 amino acids of Skp2 are sufficient to trigger degradation of the fusion protein upon inhibition of geranylgeranyl transferase I and, to a lesser extent by lovastatin (Fig. 5C). This is likely a consequence of the faster depletion of the geranylgeranylation by GGTI-298 compared to lovastatin (see above). Thus, the N-terminal region of Skp2 can function as a transferable degron in the context of GGTI-298 and lovastatin mediated Skp2 down-regulation. As this domain lacks the F-box domain and the C-terminus of Skp2, the statin-induced degradation of Skp2 does not require its incorporation into an SCF complex and therefore does not require auto-ubiquitination.

## DISCUSSION

Here we describe that the lovastatin-induced cell cycle arrest is caused by the degradation of Skp2 induced by the depletion of geranylgeranyl pyrophosphate. Skp2 is a subunit of the SCF^Skp2^ ubiquitin ligase and its statin mediated decline leads to an accumulation of the CDK inhibitors p27^Kip1^ and p21^Cip1^ and cell cycle arrest in G1 phase. The degradation of Skp2 triggered by statins is not a consequence of auto-ubiquitination or APC/C^Cdh1^-mediated degradation. Instead it is mediated by a novel pathway initiating Skp2 degradation.

Some previous studies have suggested that the accumulation of p21 and p27 may result from direct inhibition of the proteasome by lovastatin. For example, it was observed that the inactive prodrug form of lovastatin could inhibit the proteasome [22]. Mevalonate, the product of the HMG-CoA reductase reaction, could activate proteasomal activity [41], explaining why mevalonic acid releases cells from the lovastatin arrest. But a later study found no evidence that the prodrug inhibits proteasomal activity [24]. Yet a further study found that the closed ring beta-lactone form of lovastatin induced the disappearance of p27. While this closed ring form was cytotoxic, no evidence of cytostatic activity could be found [25]. This study reported in contrast that mevalonate could inhibit the trypsin like activity of the proteasome. These experiments might have been complicated by the fact that serum is a potential source of carboxyesterases that can process the prodrug to its open ring form [42].

We observed no evidence that general and off target inhibition of the proteasome activity contributes to the lovastatin mediated G1 arrest. Our data rather suggest that the inhibition of p27 and p21 degradation is substrate specific and a consequence of the ability of lovastatin to deplete geranylgeranyl pyrophosphate. This depletion triggers a novel pathway that stabilises the CDK inhibitors p21 and p27 by degradation of the ubiquitin ligase subunit Skp2. Importantly, ubiquitin-mediated degradation of p27 and p21 could be partially restored by ectopic expression of Skp2 in the presence of lovastatin (Fig. 1D), clearly demonstrating that the proteasome remains active. Similarly, deletion of the central Skp2 substrate p27 attenuated the lovastatin-induced cell cycle arrest, confirming the important role of the Skp2/p27 axis in this arrest.

Interestingly, the geranylgeranylation-dependent degradation of Skp2 is functional in different cell lines (all of those that we tested) from human or mouse, reflecting the wide-ranging cell cycle arresting activity of lovastatin previously observed. In mouse models for tumorigenesis, it has been shown that geranylgeranyl-transferase-I ablation reduces tumour formation and improves survival [43], suggesting that geranylgeranylated proteins could also be important targets of lovastatin's anti-tumour activity. Our observation that GGPP can rescue lovastatin induced Skp2 down-regulation and that inhibition of geranylgeranyl-transferase-I with GGTI-298 induces Skp2 downregulation suggests that a geranylgeranylated protein regulates Skp2 stability, whose function can be activated by inhibition of geranylgeranylation, leading to Skp2 degradation. It is tempting to speculate that this is not only a drug-specific pathway.

Geranylgeranylated Rho family GTPases, including RhoA and Rac1 have been shown to regulate G1 progression. Previous studies have suggested that geranylgeranylated Rho GTPases can destabilize p27 [44, 45] or that RhoA acts as positive regulator of cyclin E/CDK2 kinase activity [46]. Since Cyclin E /CDK2 phosphorylates p27 on T187 and this phosphorylation is required for its degradation, blocking of geranylgeranylation could inhibit p27 degradation [45, 47]. However, p27 is phosphorylated on threonine 187 in the presence of lovastatin [26] and overexpression of Skp2 can rescue p27 degradation in lovastatin-arrested cells. Other studies have suggested that the GTPase Rac1 can positively regulate Skp2 levels [48]. Inhibition of the geranylgeranylation of these small GTPases might therefore cause the decline of Skp2. However, we could not find any evidence that these GTPases or the Rho downstream kinase ROCK are involved in the statin-induced Skp2 degradation: the lovastatin/GGTI-298-induced Skp2 degradation was insensitive to the overexpression of wt or constitutively active GTPases including RhoA, Rac1A, and Rac1B; or by ROCK overexpression (JV, AM and LH, unpublished observations). In addition, lovastatin-induced Skp2 degradation was also not changed in the presence of cell permeable C3 toxin which inactivates RhoA, B and C [49].

We therefore speculate that an as yet unidentified geranylgeranylated protein is responsible for the Skp2 degradation initiated by lovastatin. However molecular details of this novel Skp2 degradation pathway remain to be elucidated in the future. Importantly, this novel pathway does not involve known mechanisms of Skp2 ubiquitin-mediated proteasomal degradation: The ubiquitin ligase APC/C^Cdh1^ is necessary and sufficient for Skp2 degradation in early G1 phase [31, 32]. We can exclude involvement of APC/C^Cdh1^ in statin-induced Skp2 degradation because lovastatin induced turnover of Skp2 also occurs in S-phase arrested cells, in which APC/C^Cdh1^ is inactive [50], and because lovastatin and GGTI-298 can induce the degradation of Skp2 in cells lacking the Cdh1 protein.

In serum-deprived cells an auto-ubiquitination mechanism regulates Skp2 stability [30, 40]. This mechanism depends on Skp2 incorporation into an SCF complex. Since we observe efficient turnover of a Skp2 mutant lacking the F-box required for the incorporation into an SCF complex and which even functions as a dominant negative protein by stabilising p27 [16], we can exclude auto-ubiquitination as a mechanism for the lovastatin/GGTI-298 induced Skp2 degradation.

We therefore conclude that lovastatin activates a novel and as yet uncharacterised pathway of Skp2 degradation. It will be interesting to determine if this pathway is only activated by drugs inhibiting geranylgeranylation, or if alternative physiological mechanisms exist in normal cells that use this pathway to induce CDK inhibitors and cell cycle arrest.

The discovery of a novel degradation pathway for Skp2 induced by statins opens up the exiting possibility that this pathway may be targeted in cancer therapy. Whilst statins have shown potential in cancer prevention, their use as a therapy may be hampered by their pleiotropic effects and tendency to accumulate in the liver. Given that Skp2 overexpression is associated with increased malignancy and poor prognosis in several types of human cancer, the future elucidation of this conserved and robust antiproliferative pathway responsible for the elimination of the Skp2 may permit the identification of novel therapeutics that could be more specific and lack the side effects of statins.

## MATERIALS AND METHODS

### Reagents

Lovastatin (MERCK #L-154, Alfa Aesar # H52792), MG132 (Sigma-Aldrich #M7479), Thymidine (Sigma-Aldrich #T9250), GGPP (Sigma-Aldrich #G6025), FPP (Sigma-Aldrich #F6892), GGTI-298 (Sigma-Aldrich #G5169). FTI-277 (Sigma-Aldrich #F9803), BrdU (Sigma-Aldrich #B9285), propidium iodine (PI) (Sigma-Aldrich #81845). Nocodazole (Sigma-Aldrich # M1404). Lovastatin was obtained in the form of an inactive beta-lactone that was activated according to the following protocol: 208mg of lovastatin was dissolved in 5ml Ethanol and then supplemented with 7.5 ml 0.1M NaOH. The solution was incubated at 50^o^C for 2 hours after which it was allowed to cool to room temperature. Finally the pH was adjusted to 7.2 after with 1M HCl. Solvent control was prepared as above but with the omission of lovastatin.

### Cell culture, transfections and lovastatin, GGTI-298, thymidine and Nocodazole treatments

The following cell lines were used in this study: Cdh1^−/−^ MEFs and U2OS cells in which Cdh1 knockdown can be induced by tetracycline (Utsh2 cells) have been described previously [51]. Cells were cultured at 37 °C with 5% CO_2_ in DMEM (HeLa S3, HeLa, Cdh1^−/−^ MEFs, Cdh1^+/+^ MEFs, NIH 3T3, p27^−/−^ MEFs, p27^+/+^ MEFs, U20S and HRT-18) or RPMI (LNCAP, K562, WM35 and U937) medium containing 10% FCS. Transient transfection experiments were conducted using Effectene® (Qiagen) transfection reagent according to the manufacturers instructions. Lovastatin was added to cells to at final concentration of 40 μM unless otherwise indicated. Treatments were carried out for the times indicated in the figure legends. GGTI-298 was similarly added to cells at a final concentration of 20 or 10 μM as indicated and incubated for the times indicated in the figure legends. FTI-277 was used at a concentration of 20μM for 36 hours. Mg132 was added together with lovastatin for 18 hours at a final concentration of 10μM. Thymidine (Sigma) was added to cells at a final concentration of 2mM. Nocodazole (Sigma) was added to cells at a final concentration of 50ng/ml

### Western blotting analysis

Cells were lysed in SDS buffer containing DTT, sonicated and incubated at 95 °C for 5 minutes. Proteins were loaded onto 13% SDS gels and resolved after which they were transferred onto PVDF membranes (Millipore) by western blotting. Bands were visualised by the ECL method (Fig. 1A, B, C, D, E; 2A, 3A-E, 4 and 5B, C) or using the Odyssey system (Licor) (Fig. 2B, C and Fig.3F).

### Generation of a Skp2 overexpressing HeLa cell line

The lentiviral Skp2 expression construct was cloned by recombining the human Skp2-cDNA (a generous gift of Wilhelm Krek) into the vector U509 (pHR-PGK-dest-bGH-polyA-SFFV-eGFP-rfa_verB generated by C. Ploner) using the Gateway® cloning technology (Life Technologies). Cells exhibiting stable Skp2 overexpression can be identified by their concomitant expression of GFP from the second promoter.

HeLa cells were transduced with lentiviral particles containing Skp2 according to the procedure previously described [52].

### Generation of of pHR-PGK-bGH-pA-SFFV-eGFP (U509)

pHR-PGK-bGH-polyA-SFFV-eGFP was generated by multicloning strategy using pHR-SIN-CSGW (kindly provided by Mary Collins, London, UK) as starting vector. pHR-PGK-SFFV-eGFP was made by amplifying the PGK-promoter from pHR-frt-PGK-PURO-frt-SFFV-eGFP (kindly provided by S. Geley, Innsbruck, Austria) using the oligonucleotides 5-'tatagaattctaccgggtaggggaggc-3′ (sense) and 5′-TATAGAATTCCATATGGTTTAAACAC GCGTTACGTAGTCGAAAGGCCCGGAGATG-3′ (antisense). The EcoRI digested PCR product was subcloned into the EcoRI site of pHR-SIN-CSGW, thereby also inserting a multiple cloning site (SnaBI-MluI-PmeI-NdeI) downstream of the PGK-promoter. In a second step, the bovine growth hormone polyadenylation sequence (bGH-pA) was PCR amplified using the oligonucleotides 5′-tataacgcgtctagagctcgctgatcagcctc-3′ (sense) and 5′-TATACATATGGATCTCGAGCCCCAGCTGG-3′ (antisense) and pHR-frt-PGK-Puro-frt-SFFV-eGFP as template. The MluI/NdeI digested PCR product was inserted into the MluI/NdeI site of pHR-PGK-SFFV-eGFP generating pHR-PGK-bGH-polyA-SFFV-eGFP. Finally this vector was rendered GATEWAY-compatible by inserting a DEST – cassette (RfA, Invitrogen, Vienna, Austria) into the SnaBI site downstream of the PGK-promoter.

### Plasmids and primers

The polymerase chain reaction was used to generate Skp2 with flanking AttB1 and AttB2. This PCR product was used in a gateway BP reaction with pDON207 GATEWAY^®^ to generate a Skp2 entry clone. This was subsequently used in a LR reaction with delta T pDEST (no tag) GATEWAY^®^ and deltaT-FLAG-DEST (NT flag tag) GATEWAY^®^ [53] to generate vectors suitable for mammalian expression. Skp2 CT and NT domain mutants were generated as described above but using primer combinations in the initial step to amplify specific domains of Skp2. To generate Skp2 lacking the first 94 amino acids, a forward primer was used that commences at nucleotide 282 in combination with a reverse primer for the full length cDNA. In contrast to generate the CT delta mutant, the full length forward primer was used together with a reverse primer starting at nucleotide 465. To generate a variant of Skp2 lacking the F-box domain we used the approach described by Carrano et al. [16]. Thus a DNA fragment of pDON207 Skp2 corresponding to nucleotides 301-946 was removed by digesting with BSpE1 and Xba1. Then a PCR fragment containing nucleotides 420-946 and flanked by BSPe1 (5′) and Xba1 (3′) sites respectively was ligated into the cut vector generated a mutant lacking nucleotides 301-419, which correspond to the F-box region of the Skp2 protein.

To generate a Skp2 with a C-terminal eYFP fusion to PCR reactions were performed to amplify Skp2 with a 5′ attB1 site and a 3′ Spe1 restriction site and to amplify eYFP with a 5′ Spe1 site and a 3′ attB2. After Spe1 digestion of the resulting PCR products, a ligation was performed to generate Skp2 fused to eYFP with flanking attB1 and attB2 sites. This was used in a BP reaction and transferred into a Delta T – Flag pDEST for mammalian expression.

### Northern blotting

Total RNA was isolated from HeLa cell pellets using the RNeasy Mini kit (Qiagen). The RNA was then subjected to electrophoretic separation using a 1% (wt/vol) agarose-formaldehyde (0.75% v/v) gel after which it was transferred to a positively charged Hybond membrane (GE healthcare). The membrane was UV irradiated in order to fix the RNA. ^32^P-labeled DNA probes for Skp2 and GAPDH were prepared using the Random primer DNA labeling system (Invitrogen) using restriction digest fragments of Skp2 and GAPDH cDNA. Hybridization was performed by incubating the membrane in ULTRAhyb® Ultrasensitive Hybridization Buffer (Ambion) containing the probe at 42 °C for 16 hours. After washing bands were visualized by autoradiography and quantification performed by phosphorimaging analysis.

### Antibodies

Antibodies. The following antibodies were used in this study. Skp2 (Rabbit polyclonal H-435, Santa Cruz # sc-7164, 1:500), Skp2 (Mouse monoclonal A2, Santa Cruz #sc-74477 1:250), p27 (Mouse monoclonal, BD Transduction laboratoriesn#610242,1 in 250), p27 (rabbit polyclonal, C19 # sc-528, SantaCruz), p21 Mouse (Mouse monoclonal anti-human Cip1, BD Transduction laboratories, #610234), 1 in 250) p57 (C-20, Santa Cruz,sc-1040) p16 INK4a (Mouse monoclonal, G175-405, Pharmingen # 13251A, 1in 100), p15 Ink4b (Rabbit polyclonal, K-18, Santa Cruz, # sc-613) Cyclin E (mouse monoclonal HE12, Santa Cruz), Cyclin A (Polyclonal rabbit serum, produced in Hengst laboratory), PSTAIRE (Murine cell supernatant, Cdk4 (Rabbit Polyclonal, C-22, Santa Cruz, sc-260), Tubulin (mouse monoclonal, b-tubulin AA2 Sigma-Aldrich T8328), Cdh1/Fizzy related (homemade, rabbit 1 in 100), GAPDH mouse monoclonal (6C5, Calbiochem 1in 1000), Actin (I-19, Santa Cruz), GFP (Mouse Mixture of two monoclonal antibodies (7.1 and 13.1) Roche, # 11814460001, 1in 1000) Flag (mouse monoclonal, M2 Sigma Aldrich #F3165).

### FACS

Cell cycle distribution was determined by using BrDU / propidium iodide double staining. Briefly, 20μM BrdU was added to cells approximately 1 hour before harvest. Cells were fixed in a 70% ethanol solution and after denaturation of DNA incubated with FITC labelled anti BrdU antibody and 0.5ug/ml propidium iodide. Cells were analysed using a BECKMANN COULTER EPICS XL MCL Flow Cytometer and cell cycle distribution determined using FlowJo cytometry data analysis software software.

## SUPPLEMENTARY MATERIAL, FIGURES


