# Supplementary material for: Statin-induced depletion of geranylgeranyl pyrophosphate inhibits cell proliferation by a novel pathway of Skp2 degradation
Source: Oncotarget. 2014 Dec 26;6(5):2889–902. doi: 10.18632/oncotarget.3068 (PMC4413625; doi:10.18632/oncotarget.3068)
Supplement: Supplementary file 1 [file oncotarget-06-2889-s001.pdf]

Statin-induced depletion of geranylgeranyl pyrophosphate inhibits cell proliferation by a novel pathway of Skp2 degradation

Supplementary Material

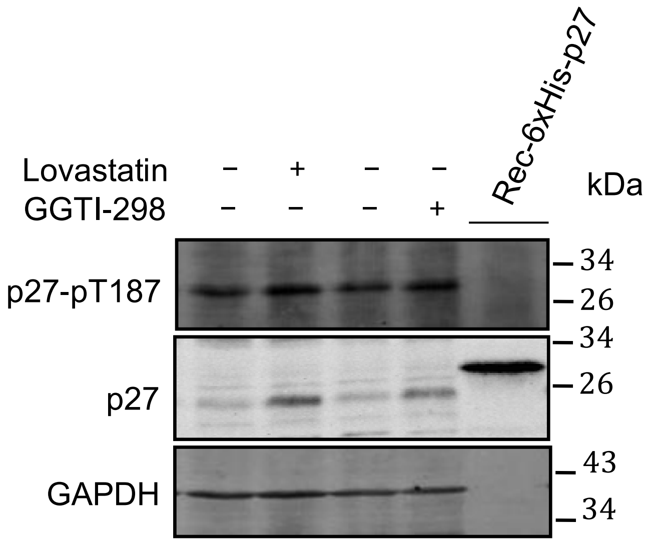

Supplementary figure 1: threonine 187 phosphorylated p27 is expressed in lovastatin or GGTI-298 treated cells.

HeLa cells were treated with either 40 µM lovastatin (lane 2) or 10 µM GGTI-298 (lane 4) for 24 hours and analysed by immunoblotting using a monoclonal antibody specific for threonine 187 phosphorylated p27 (clone 2B10B7, life technologies), p27 (polyclonal antibody, C19, Santa Cruz), and GAPDH (Calbiochem, clone 6C5) as a loading control. Purified recombinant hexahistidine-tagged p27 was also separated (lane 5) to confirm the specificity of the antibody.
